# Flight-Fecundity Trade-offs: A Possible Mechanistic Link in Plant–Herbivore–Pollinator Systems

**DOI:** 10.3389/fpls.2022.843506

**Published:** 2022-04-25

**Authors:** Goggy Davidowitz, Judith L. Bronstein, Natasha Tigreros

**Affiliations:** ^1^Department of Entomology, University of Arizona, Tucson, AZ, United States; ^2^Department of Ecology and Evolutionary Biology, University of Arizona, Tucson, AZ, United States

**Keywords:** pollination, herbivory, nutrient tradeoffs, Lepidoptera, nuptial gift, nectar

## Abstract

Plant–herbivore and plant–pollinator interactions are both well-studied, but largely independent of each other. It has become increasingly recognized, however, that pollination and herbivory interact extensively in nature, with consequences for plant fitness. Here, we explore the idea that trade-offs in investment in insect flight and reproduction may be a mechanistic link between pollination and herbivory. We first provide a general background on trade-offs between flight and fecundity in insects. We then focus on Lepidoptera; larvae are generally herbivores while most adults are pollinators, making them ideal to study these links. Increased allocation of resources to flight, we argue, potentially increases a Lepidopteran insect pollinator’s efficiency, resulting in higher plant fitness. In contrast, allocation of resources to reproduction in the same insect species reduces plant fitness, because it leads to an increase in herbivore population size. We examine the sequence of resource pools available to herbivorous Lepidopteran larvae (maternally provided nutrients to the eggs, as well as leaf tissue), and to adults (nectar and nuptial gifts provided by the males to the females), which potentially are pollinators. Last, we discuss how subsequent acquisition and allocation of resources from these pools may alter flight–fecundity trade-offs, with concomitant effects both on pollinator performance and the performance of larval herbivores in the next generation. Allocation decisions at different times during ontogeny translate into costs of herbivory and/or benefits of pollination for plants, mechanistically linking herbivory and pollination.

## Introduction

Plant–herbivore and plant–pollinator interactions are both well-established, but largely independent fields of study. Pollination is a mutually beneficial interaction and historically has been the most thoroughly studied of all mutualisms (Bronstein, 1994). The key issue in the study of pollination is how plants obtain and donate high-quality pollen to maximize reproductive output. In the case of the over 85% of plant species that are animal-pollinated (Ollerton et al., 2011), this involves attracting and rewarding partners that will transfer pollen among flowers of the same species. Herbivory, in contrast, is an antagonistic interaction between plants and animals. In some cases, consumption of leaves can dramatically reduce plant growth and survival (Lehndal and Ågren, 2015). Key issues in the study of herbivory have been how plants defend themselves against being eaten, and when and how herbivores are able to circumvent these defenses (Núñez-Farfán et al., 2007).

In recent years, it has become increasingly well-recognized that pollination and herbivory are not, as might be suggested by these contrasting concerns, independent of each other (Rusman et al., 2019). Rather, they interact in ways that synergistically contribute to a plant’s reproductive success (Marquis, 1992; Bronstein et al., 2007; Jacobsen and Raguso, 2018; Haas and Lortie, 2020; Johnson et al., 2021). The presence of herbivore damage, for instance, can reduce the likelihood that pollinators will be attracted to flowers; it can also reduce resources necessary to produce flowers, seeds, and fruits. Herbivores may also simply consume the flowers. In all of these cases, herbivory reduces plant fitness through reduced effectiveness of pollination. In other situations, however, the presence of herbivores actually enhances pollination. This occurs, for example, when a single species is both the pollinator and herbivore of the same plant species. In these cases, the probability of pollination and herbivory increase together. The best-known examples are highly specialized insects, such as fig wasps and yucca moths, that pollinate plants, then lay eggs in the flowers, with the pollinator’s offspring subsequently destroying a portion of the developing seeds (Kato and Kawakita, 2017). More common, but not as well-studied, are cases in which insects feed on floral nectar, then lay eggs on the leaves of the same individual plant or on neighboring plants of the same species; the pollinator’s offspring in this case are folivores of their host plant. The best-known of these herbivorous pollinators are Lepidoptera, including but not restricted to those with narrow diet breadths (Bronstein et al., 2009; Altermatt and Pearse, 2011).

Recent conceptual advances linking herbivory and pollination have largely adopted a plant perspective (e.g., Lucas-Barbosa, 2016; Jacobsen and Raguso, 2018; Kessler and Chautá, 2020). In this perspective, we develop a framework that links herbivory and pollination from the animal perspective instead. Specifically, we explore the idea that trade-offs between investment into flight vs. fecundity functionally link insect pollination and herbivory. Flight–fecundity trade-offs in insects are a well-studied phenomenon (Johnson, 1963; Roff, 1986, 1990, 1994; Rankin and Burchsted, 1992; Dingle, 1996; Zera et al., 1999; Zera and Brink, 2000; Zera and Larsen, 2001; Gu et al., 2006; Hanski et al., 2006; Karlsson and Johansson, 2008; Guerra and Pollack, 2009; Tigreros and Davidowitz, 2019). At a basic level, allocation of resources to flight will modify an insect pollinator’s efficiency, with a resultant increase in plant fitness. In contrast, allocation of resources to fecundity leads to an increase in the herbivore population size produced in the next generation.

Increased allocation of resources to fecundity may or may not translate linearly into herbivore damage as damage may differ among populations (Marquis, 1992), the strength of selection induced by the herbivore can differ (Agrawal et al., 2012), tolerance vs. resistance to herbivores may mitigate damage (McCall et al., 2020), when during ontogeny herbivory occurs effects overall damage (Boege and Marquis, 2005) and the quality of the host plant and its effect on herbivore growth may mitigate damage (Davidowitz et al., 2003; Wilson et al., 2019), among other factors.

Larval Lepidoptera are predominantly herbivores and most adults are pollinators (Hahn and Brühl, 2016), often of the same plant species (Altermatt and Pearse, 2011), making them ideal to address this link between herbivory and pollination. We note that this linkage exists whether the pollinator lays eggs on the same plant or on different individual plants of the same species and whether the plant being eaten and the plant being pollinated are different species, which may result in differential costs and benefits of herbivory and pollination, respectively.

Here, we associate resource allocation to flight with increased pollination efficiency and allocation to fecundity with herbivory damage. In addition to nectar foraging and pollen transfer, flight is of course also used for other functions, such as to find mates and host plants (Chai and Srygley, 1990; Willis and Arbas, 1991; Mitra et al., 2016). However, because nectar foraging is the most relevant function of flight to a plant’s fitness due to its resultant pollination, we focus on the nectar foraging function of flight.

The efficiency of an animal as a pollinator entails more than just flight. It encompasses numerous pollination-related traits including multimodal signaling, used by the pollinator to find the flower (Raguso and Willis, 2002), the reliability of the signal used by the plant to attract the pollinator (Von Arx et al., 2012), proboscis length matching with nectar tube length (Haverkamp et al., 2016; Soteras et al., 2020), flower handling time (Kunte, 2007; Riffell and Alarcón, 2013), pollen transport distances (Herrera, 1987), and floral constancy (Goulson et al., 1997). We focus on allocation to flight (flight muscles and wings), as this is the largest resource sink related to pollination (G. Davidowitz, unpublished data).

Below, we first provide a general background on trade-offs between flight and fecundity in insects. We then examine the sequence of resource pools available to Lepidopteran herbivores and pollinators. Finally, we discuss how subsequent acquisition and allocation of resources from these pools may alter the flight–fecundity trade-off, with concomitant effects both on pollinator performance and the performance of larval herbivores in the next generation.

## Flight–Fecundity Trade-Offs

In insects, allocation to flight begins with an allocation to flight muscle and wings: larger flight muscles increase power output and larger wings reduce wing loading, both of which increase flight performance (Dudley, 2002). In general, resource allocation to flight is essential as it allows the adult to find mates, disperse, and forage for additional resources. In insect pollinators in particular, the dimensions of flight muscle and wings can have significant effects on pollinator flight (Dudley, 2002), affecting, for example, the ability to forage for nectar from flowers buffeted by the wind while hovering (Hedrick and Daniel, 2006; Sprayberry and Daniel, 2007). Subsequent investments are needed to fuel flight itself, which is the most energetically expensive mode of locomotion known (McCallum et al., 2013). In insects, flight can be 30-fold more costly than terrestrial locomotion (Harrison and Roberts, 2000). Insects that act as pollinators often hover while feeding on nectar, a behavior that is energetically demanding (Biewener and Patek, 2018). For example, hovering hawkmoths require 170 times more energy than basal metabolism (Bartholomew and Casey, 1978). The energy from nectar available to the insect differs across plant species and may differ among plant populations and communities as well (Nicolson et al., 2007; Lebeau et al., 2016).

The nectar load itself can affect the stability and maneuverability of the insect in flight, with potential effects on feeding efficiency (Mountcastle et al., 2015). Feeding efficiency, in turn, may translate into pollinator effectiveness (Goulson, 1999). Flight distance is an important component of pollinator efficiency as it may affect the pollen dispersal ability of the insect pollinator (Schulke and Waser, 2001; Pasquet et al., 2008).

Allocation to reproduction involves investments into the reproductive system as well as to eggs. Larval diet can affect the number of ovarioles in the ovary, and hence the maximum number of eggs that can be laid; fecundity is reduced on poor quality larval diets due to fewer ovarioles (Sisodia and Singh, 2012; Aguila et al., 2013). In all insects, reproductive output is determined by the availability of nutritional resources, whether acquired during the larval or the adult stages (Wheeler, 1996; Papaj, 2000; Awmack and Leather, 2002). This is discussed in depth, below.

Investments in flight and fecundity trade off (two words) because both require the same macronutrient resources, proteins, carbohydrates, and lipids, all of which are often in limited supply (Baker and Baker, 1986; van Noordwijk and de Jong, 1986; Stearns, 1989; Zera and Harshman, 2001; Boggs, 2009; Saeki et al., 2014; Tigreros and Davidowitz, 2019). Although other limiting resources, such as time available to devote to life-history activities, can also trade-off, nutrient-based trade-offs are probably the dominant type of trade-off in nature (Zera and Harshman, 2001; Boggs, 2009; Agrawal, 2020). Tigreros and Davidowitz (2019) showed that in wing monomorphic insect species, 76% of studies showed a flight–fecundity trade-off when resource availability was manipulated. The more resources allocated to flight, the fewer resources that are available for fecundity (and vice versa), resulting in a negative association between flight and fecundity. As a consequence, we can predict a negative association between the role of an insect as an herbivore and that as a pollinator (see above). With this introduction to nutrient-based trade-offs between flight and fecundity, we next examine the sequence of nutrient pools available to Lepidoptera.

## The Sequence of Resource Pools

**Figure 1:**
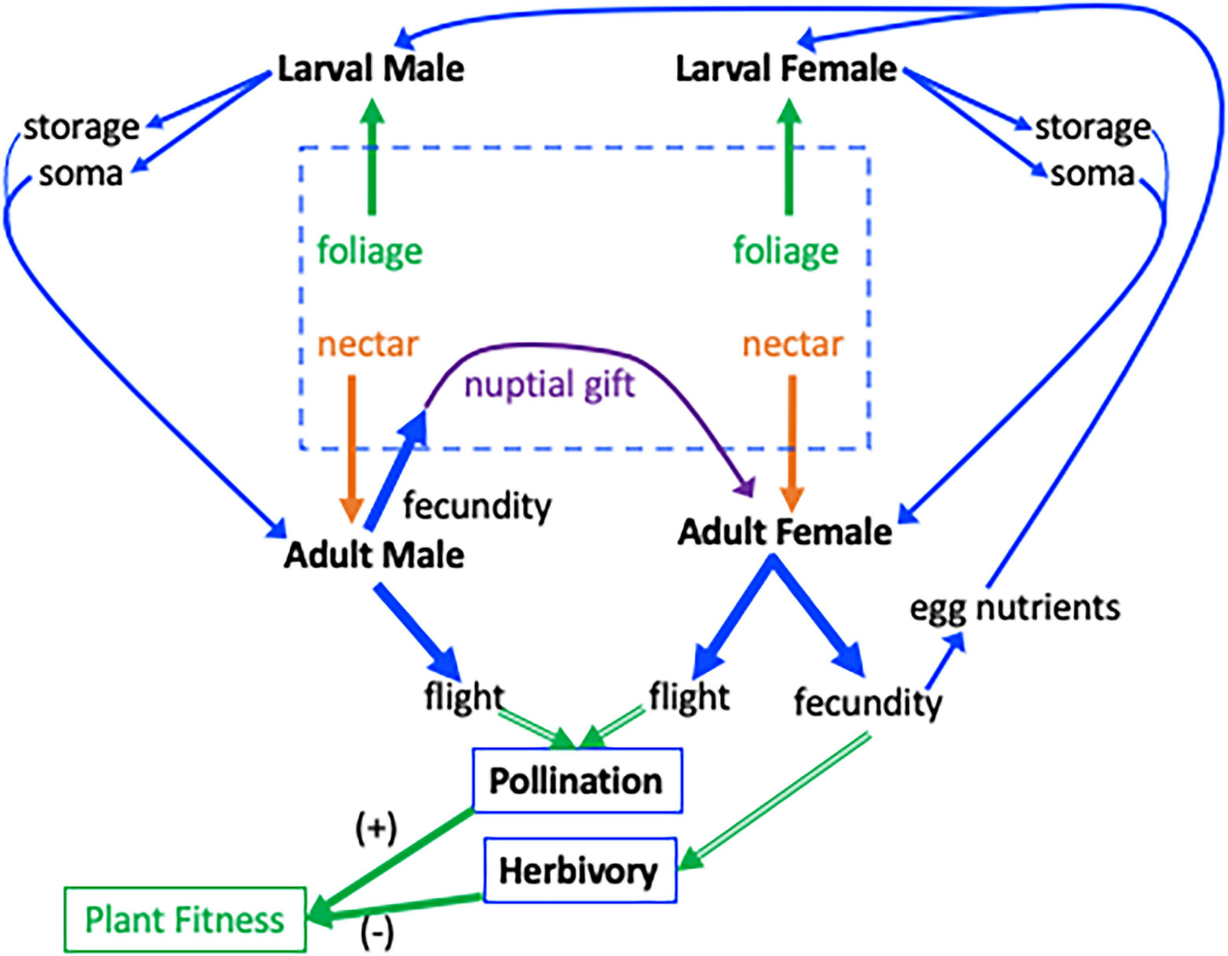
Interaction between a plant and a Lepidopteran that is an herbivore as a larva and a pollinator as an adult. The central dashed box indicates resource pools to the insect. Host-plant foliage is the resource for larvae (green arrows from dashed box), nectar is a resource for adults (orange arrows), and nuptial gifts are a resource given to the female by the male (purple arrow). For simplicity, only resources relevant to flight–fecundity trade-offs are shown and allocation to other functions such as maintenance, are omitted. Blue lines indicate resources and green lines indicate effects on plant fitness. Larvae consume foliage for nutrient storage and growth (soma; strait blue arrows at top) which are available as resource pools in the adult following metamorphosis (curved blue arrows). Adult Lepidoptera can allocate resources to flight or fecundity (thick blue arrows). The consequences of flight–fecundity allocation decisions to the plant (double-lined green arrows) through herbivory and pollination are indicated by the thick green arrows. Allocation of resources to fecundity by males and females reduces plant fitness, green arrow (−), *via* herbivory. Allocation of resources to flight increases plant fitness, (+) green arrow, through pollination. Eggs produced by male allocation to nuptial gifts, and female allocation to fecundity, produce the next generation of herbivores (rightmost blue arrow).

The timing of the acquisition and allocation of nutrients can influence acquisition of additional resources (Figure 1). Some empirical studies suggest that allocation to traits related to acquisition ability, such as flight, may directly influence the further acquisition of resources (King et al., 2011; Descamps et al., 2016). Increased allocation to locomotion, for example, can improve an organism’s ability to forage and acquire additional resources. The quantity and quality of resources that a juvenile herbivore acquires can modify its nectar preferences as an adult (Mevi-Schütz and Erhardt, 2003); this in turn may influence its effectiveness as a pollinator.

We distinguish between plant-derived resources (foliage and nectar) and insect-derived resources (maternally provided provisions to the egg, and nuptial gifts that males provide to females during copulation). These resources are available at different times during an insect’s ontogeny (Figure 1) and differ in their relative amounts of proteins, carbohydrates, and lipids (see below). These resource pools can have significant consequences for the growth of the herbivorous juvenile and the pollinating adult, with potential fitness consequences to the plant. Below, we examine each of these resource pools in the order they are available to the insect.

## Resource Acquisition and Allocation in Herbivorous Juveniles

### Maternally Provisioned Resources

The first resource pool to which herbivorous insects have access is provided by mothers, through the nutritional resources they deposit into eggs (Roach and Wulff, 1987; Bernardo, 1996; Fox and Czesak, 2000). In contrast to the leaf tissue that will be consumed once the insect emerges from the egg (see below), nutrients in eggs include substantial amounts of proteins (~40%–50%) and lipids (30%–40%). As a consequence, maternal egg provisioning of nutritional resources can have profound effects on offspring development and subsequent life-history traits (Mousseau and Dingle, 1991; Bernardo, 1996; Mousseau and Fox, 1998; Fox and Czesak, 2000; Hunt and Simmons, 2000). This in turn can influence flight–fecundity trade-offs once the offspring eclose as adults. At the same time, females experiencing flight–fecundity trade-offs may adjust the number of eggs they produce as well as the quantity of nutrients provisioned to each egg (Tigreros and Davidowitz, 2019). Females of the Speckled Wood butterfly, *Pararge aegeria,* that are forced to fly long distances, for example, produce smaller eggs and smaller offspring that take longer to develop (Gibbs et al., 2010). Similarly, females experiencing poor nutritional environments during either the larval or adult stage generally decrease the nutrients they put into eggs (Bernardo, 1996; Mevi-Schütz and Erhardt, 2005; Geister et al., 2008). In other cases, however, Lepidoptera may increase nutrient investment in eggs to improve offspring performance on low-quality host plants (Rotem et al., 2003). As a consequence, the provisioned egg itself may provide a link between the maternal and offspring resource acquisition and allocation strategies, as well as associated life-history trade-offs (Figure 1).

### Leaf Tissue

The larvae of most Lepidoptera feed on green plant tissues. These tissues contain large amounts of carbohydrates, but only a small fraction of the lipids and protein (nitrogen) that a larva needs. While some of the dietary carbohydrates are converted into lipids (Arrese and Soulages, 2010), the limited availability of dietary protein leads to a fundamental nutritional mismatch between Lepidoptera (as well as other herbivores) and their host plants (Slansky, 1978; Mattson, 1980; Wilson et al., 2019). For example, host plants of the cabbage butterfly (*Pieris rapae*) contain only 1.9%–5.9% N (~9.4%–36.9% protein), compared to about 13% N content in the adult bodies at eclosion (Morehouse and Rutowski, 2010). To make up such differences, insects engage in compensatory feeding, eating more of nutrient-poor diets to reach their nutritional requirements (Simpson and Simpson, 1990; Nestel et al., 2016). This nutritional mismatch in the larval stage often contributes to flight–fecundity trade-offs in Lepidoptera, because limited nutritional resources from leaf tissue are differentially allocated to flight (wings and flight muscle) vs. reproductive (ovaries and eggs) structures of the adult (Tigreros and Davidowitz, 2019). Furthermore, some of the resources acquired from the larval diet are stored and carried over through metamorphosis (Arrese and Soulages, 2010). After emerging, but before finding a nectar source, adults must maintain their bodies and fuel flight solely with larval stores. These endogenous reserves can be used, together with adult feeding, to produce eggs and fuel flight (Figure 1).

Two contrasting scenarios of allocation of nutrients from leaf tissue can be envisioned. First, when juvenile resources are limited, due either to low abundance or to low nutritional value of the host plant, fewer resources will be available to “build” the adult. In one scenario, we hypothesize that fewer resources are allocated to flight but allocation to fecundity is maintained, resulting in reduced efficiency of the adults during the feeding stage (when pollination occurs), while maintaining a high level of offspring herbivory. A net reduction in plant fitness might result. Alternatively, in a second scenario, we hypothesize that reduced nutrients available for juvenile herbivores may result, in the adult stage, in reduced allocation of resources to fecundity but not to flight. In this case, pollination efficiency may remain high and herbivore populations may be smaller in the next generation, with net fitness benefits to the plant.

## Resource Acquisition and Allocation in Pollinating Adults

### Floral Nectar

Nutrient deficiencies in the larval stage, which, can lead to flight–fecundity trade-offs, might be compensated for by the subsequent acquisition and allocation of nectar nutrients (Figure 1). A growing number of studies indicate that nectar can be as important as larval-derived reserves in supporting both flight and fecundity in adult females. Throughout their adult lives, moths and butterflies typically feed on floral nectars, which are carbohydrate-rich solutions (20%–50% sugars) enriched by small amounts of essential and non-essential amino acids (Baker and Baker, 1986; Lanza et al., 1995; Nicolson and Thornburg, 2007; Willmer, 2011). In general, females that feed on nectar produce more eggs than females that do not (Sasaki and Riddiford, 1984; von Arx et al., 2013). There are at least two explanations for this. First, carbohydrates from nectar provide the energy necessary to fuel flight (O’Brien, 1999), and contribute to the synthesis of non-essential amino acids for egg production (O’Brien et al., 2002, 2004). Second, contrary to the paradigm that essential amino acids can only be drawn from the larval diet (O’Brien et al., 2002), some studies have shown that nectar-derived essential amino acids enhance fecundity in Lepidoptera (Mevi-Schütz and Erhardt, 2005; Levin et al., 2017b), especially when resources acquired by the larvae are limited (Mevi-Schütz and Erhardt, 2005).

Resources acquired by male and female adult Lepidoptera (and other nectar-feeders) are not necessarily identical. In a comprehensive literature review, Smith et al. (2019) showed that male and female pollinators differ in the species of flowers visited, as well as in their visitation frequencies. Female pollinators tend to visit a higher diversity of flowers than males, whereas males tend to forage over greater distances than females. These differences can potentially result in differences between conspecific males and females in their quality as pollinators (Smith et al., 2019). Once nectar has been ingested, how it is subsequently invested into life-history functions can also differ between sexes: females metabolize nectar-derived amino acids before utilizing larval-derived amino acids, whereas males preferentially use amino acids from larval stores before using those derived from nectar (Levin et al., 2017a). Males also allocate more nectar-derived amino acids to flight muscles than do females (Levin et al., 2017a). Finally, there are sex-related differences in how essential (EAA) and non-essential amino acids (NEAA) are allocated: after feeding, males metabolize EAAs more readily than females, whereas females preferentially allocate EAAs to reproduction (Levin et al., 2017a).

### Male Nuptial Gifts

Adult females can acquire nutrients from nuptial gifts, not only from nectar. These nutritional gifts are a type of reproductive investment that is widespread across animal taxa (Vahed, 1998; Lewis and South, 2012; Boggs, 2018). In insects, males transfer a structure called a spermatophore during mating, which includes both sperm and additional nutrients. These nutrients can be used by the female in oogenesis and somatic maintenance (Boggs, 1990, 1997; Karlsson, 1998). In contrast to leaf tissue and nectar, nuptial gifts contain substantial amounts of protein. For example, nuptial gifts in Pierid butterflies contain as much as 50% protein (Bissoondath and Wiklund, 1996; Karlsson, 1998; Tigreros, 2013) with a large percent of that being essential amino acids: for example, ~35% (Meslin et al., 2017). While providing an additional source of macronutrients for adult females, nuptial gifts have the potential to both ameliorate and magnify flight–fecundity trade-offs. In Pierids, a single nuptial gift can provide the necessary nutrients to produce 50–80 eggs, a substantial contribution to female fecundity (Karlsson, 1998; Wiklund et al., 1998; Wedell and Karlsson, 2003). Amino acids supplied through nuptial gifts can change female reliance on amino acid-rich nectar preference (Mevi-Schütz and Erhardt, 2003), which may affect the pollination efficiency of the female. At the same time, because a nuptial gift is more than 80% water (Boggs and Watt, 1981), an important resource in arid environments (Contreras et al., 2013), female acquisition of nuptial gifts can increase the cost of flight by increasing wing loading. For example, a fresh spermatophore in *P. rapae* may add up to 10% of the female eclosion mass (Tigreros, unpublished).

Males may rely on both larval- and adult-derived resources to produce nuptial gifts. For example, nitrogen content in larval diets can change the composition of nuptial gifts (Bonoan et al., 2015), and nectar uptake by males can increase the size of the nuptial gift by adding more nutrients than those derived from the larva diet (Watanabe and Hirota, 1999; Levin et al., 2016). Nuptial gifts can be costly to produce, representing up to 15% of the male body weight in Lepidoptera (Svärd and Wiklund, 1989). As a consequence, males of species with substantial nuptial gift donation may prefer to mate with (Rutowski, 1985; Tigreros et al., 2014), and transfer more nutrients to females that are more fecund (Bonoan et al., 2015). In this case, a female’s ability to acquire nutrients from this resource pool (Tigreros, 2013; Tigreros et al., 2014; Bonoan et al., 2015) would depend on how she had previously allocated resources to flight and fecundity (Figure 1).

## The Effects of Sequential Acquisition and Allocation of Resources on Plant Fitness

The acquisition of resources has typically been considered as a single event (the stem of the “Y” model, *sensu* van Noordwijk and de Jong, 1986). In most systems, however, resource acquisition and decisions governing resource allocation are not fixed, but rather dynamic processes that change continually across an organism’s life (Zera and Harshman, 2001; Boggs, 2009; Kooijman, 2009; Figure 1). Acquisition of additional resources is predicted to reduce or mask potential trade-offs (Kaitala, 1987; Chippindale et al., 1993; Nijhout and Emlen, 1998; Zera and Harshman, 2001; Harshman and Zera, 2007). This suggests that organisms may have a means to modulate (and even ameliorate) the expression of a trade-off when acquiring resources from additional pools, with implications for plant fitness. For example, females of the Map butterfly, *Araschnia levana,* raised on low-quality larval diets prefer nectar with amino acids, whereas females raised on high-quality diets do not (Mevi-Schütz and Erhardt, 2003). These nectar amino acids can enhance butterfly fecundity thereby increasing damage by the offspring herbivores (Mevi-Schütz and Erhardt, 2005). Thus, the sequential acquisition of resources may change their allocation to flight or to fecundity over time.

Therefore, we may also expect the strength of the trade-off between flight and fecundity to change as the nutritional needs and nutrient availability change across an organism’s life cycle (Figure 1). For example, an herbivore feeding on a nutritionally poor host plant might allocate more resources to flight at the expense of fecundity, with the potential fitness benefit to the plant. If, however, the emerged adult has access to an abundance of nutrient-rich nectar, it may shift these resources to increased fecundity (Sasaki and Riddiford, 1984; Levin et al., 2016, 2017a), thereby obviating the flight–fecundity trade-off imposed by larval resources. In another example, resources already allocated to flight may be reallocated to reproduction following flight muscle histolysis in aging butterflies (Jervis et al., 2005; Stjernholm et al., 2005), with a resultant increase in herbivory costs to the plant.

## Future Directions

In this perspective, we have argued that trade-offs in resource allocation between flight and fecundity in insects can provide a mechanistic link between pollination and herbivory with subsequent effects on plant fitness. To further develop this idea, we provide additional questions for future research.

1. Here, we have focused on Lepidoptera. Do flight fecundity trade-offs in other insect pollinator taxa, such as solitary bees, flies, and beetles, have similar effects on plant fitness?
2. We have argued that flight–fecundity trade-offs should have a direct impact on plant reproduction. It will be exciting to explore, *via* models and empirical studies, how flight–fecundity trade-offs influence plant population dynamics and evolution. Do different strengths of these trade-offs translate to different effects on the plants?
3. We have focused on insects that feed on leaves as juveniles and on nectar as adults. However, some specialized insect pollinators feed on seeds in the juvenile stage; still others shift from feeding on leaves to feeding on flowers when the latter become available. In many cases the adults do not feed at all (e.g., fig wasps and yucca moths; Kato and Kawakita, 2017). Do the flight–fecundity trade-offs discussed here illuminate these interactions as well?
4. In arid environments, water is another critical resource that adult insects gain from feeding on nectar (Contreras et al., 2013). Does this additional resource alters in any way the resource allocation trade-offs between flight and fecundity we discuss here?
5. Does plant density-dependence affect how the flight–fecundity trade-off affects plant fitness? More specifically, does the flight–fecundity trade-off differentially affect pollination when the pollinator has numerous, vs. few, plants available at which it can feed, and how does the flight–fecundity trade-off affect herbivory when the female can lay eggs on numerous versus few possible host plants?

These, and additional, yet to be identified questions, make flight–fecundity trade-offs an exciting area of future research into the mechanistic link between pollination and herbivory, and plant–insect interactions more broadly.

## Data Availability Statement

The original contributions presented in the study are included in the article/supplementary material; further inquiries can be directed to the corresponding author.

## Author Contributions

GD, NT, and JB developed the ideas for the manuscript and all authors were involved in the writing and editing. All authors contributed to the article and approved the submitted version.

## Funding

This work was supported by National Science Foundation (NSF-USA) grant IOS-2122282 to GD and NT.

## Conflict of Interest

The authors declare that the research was conducted in the absence of any commercial or financial relationships that could be construed as a potential conflict of interest.

## Publisher’s Note

All claims expressed in this article are solely those of the authors and do not necessarily represent those of their affiliated organizations, or those of the publisher, the editors and the reviewers. Any product that may be evaluated in this article, or claim that may be made by its manufacturer, is not guaranteed or endorsed by the publisher.
